# Items

**Published:** 1882-02

**Authors:** 


### Items.

OBITUARY.—Died, at his residence near Philadelphia, on the 31st of October, 1881, William Furness Jenks, M.D., aged thirty-nine years.

The death of Dr. Jenks finished a life which a few years ago was full of promise. Educated at Harvard, he received his medical degree from the University of Pennsylvania in 1866, and after a term of service as resident physician in the Philadelphia Hospital, he studied obstetrics and gynæcology in the special schools of Berlin, Vienna, London and Edinburgh. In 1870 he began the practice of his profession in Philadelphia, and lost no time in finding in dispensary service the opportunity of acquiring a practical knowledge of the sciences which he had so carefully studied, and for three years gave a course of practical obstetrics, illustrated by operations on the cadaver. For only a few years, however, was he able to continue his work. In the autumn of 1874, failing health compelled him to pass the winter in Florida. On his return, he made an effort to resume work, but was soon forced to give up, and again seek in a foreign climate the health refused him in his own. From this time his professional labors were over, for he saw that the only hope of prolonging his life was in the entire abandonment of all work. For a few years his search for health was partially successful, and he lived in comparative comfort until last October, when, after a short confinement in bed, he quietly passed away.

Breaking down almost at the beginning of his professional life, Dr. Jenks left but little behind him by which he could be judged, and the full measure of our loss properly estimated. His exhaustive review of Schrœder (*American Journal Med. Sci-*

*ences, Oct.*, 1872), and the paper on "Sarcomatous Growths of the Uterus," (*Amer. Supplement to the Obs. Journ. of G. B. and I., Oct. and Nov.* 1873), bear, however, sufficient evidence of the extent of his knowledge, and of the close analytical character of his mind. A pupil of Sir James Y. Simpson, he regarded the obstetric forceps as extractors, rather than compressors, and after persistent efforts, succeeded in introducing the Simpson Forceps into Philadelphia obstetric practice. A skillful pathologist, he, at the time of his first illness, was preparing a course of lectures on uterine and ovarian pathology for the College of Physicians. Had he been able to complete this course, it would have been a very valuable contribution to this branch of medical science. It, however, was not to be, and when he died, almost nothing remained to his friends, save the recollection of the brilliancy of his genius, his patient industry, and the sparkling geniality of his manner.—*The Medical News and Abstract, December*, 1881.

———

THE SIXTEEN COMMANDMENTS OF THE PARIS ACADEMY OF MEDICINE.—Translated by Dr. D. C. Holiday, for the *New Orleans Med. and Surg. Journal.*

The Academy of Medicine has condensed into the following sixteen propositions the most important hygienic rules for the care and management of infants. We reproduce them here, with the sincere hope that all mothers and nurses will commit them to memory and observe them as faithfully as the ten commandments of holy writ:

I. During the *first year* the only suitable nourishment for an infant is its own mother's milk, or that of a healthy wet-nurse. Suckling should be repeated every *two* hours—*less* frequently at night.

II. When it is impossible to give breast-milk, either from the mother or a suitable nurse, cow's or goat's milk given tepid, reduced at first one-half by the addition of water slightly sweetened, and after a few weeks one-fourth only, is the next best substitute.

III. In giving milk to an infant always use glass or earthenware vessels, not metallic ones, and always observe the most scrupulous cleanliness in their management, rinsing whenever used. Always avoid the use of teats of cloth or sponge, so frequently used to appease hunger or quiet crying.

IV. Avoid carefully all those nostrums and compounds so liberally advertised as superior to natural food.

V. Never forget that artificial nourishment, whether by *nursing-bottle* or *spoon* (without the breast), increases to an alarming degree the chances of producing sickness and death.

VI. It is always dangerous to give an infant, especially during the first two months of its life, solid food of any kind—such as bread, cakes, meats, vegetables or fruit.

VII. Only after the *seventh* month, and when the mother's milk is not sufficient to nourish the child, should *broths* be allowed. After the first year is ended, then it is appropriate to give light broths or paps, made with milk and bread, dried flour, rice, and the farinaceous articles, to prepare for weaning. A child ought not to be weaned until it has cut its first twelve or thirteen *teeth*, and then only when in perfect health.

VIII. An infant should be washed and dressed every morning before being nursed or fed. In bathing a child temper the water to the weather, carefully cleanse the body, and especially the genital organs, which require great cleanliness and care; and the head should be carefully freed from all scabs and crusts which may form. Where the belly-band is used it should be kept on for at least one month.

IX. An infant's clothing should always be so arranged as to leave the limbs freedom of motion, and not to compress any portion of the body.

X. An infant's clothing should be studiously adapted to the weather, avoiding at all times exposure to the injurious effects of sudden changes in the temperature without proper covering; but nurseries and sleeping apartments should invariably be well ventilated.

XI. An infant should not be taken into the open air before the fifteenth day after birth, and then only in mild, fair weather.

XII. It is objectionable to have an infant sleep in the same bed either with its mother or nurse.

XIII. No mother should be in too great a hurry to have a child walk; let it crawl and accustom itself to rising on its feet by climbing on articles of furniture, or assisted by the arms of a careful attendant. Great care should be taken in the too early use of baby-wagons, etc.

XIV. No trifling ailments in infants, such as colics, frequent vomiting, diarrhœa, coughs, etc., if persistent, should be neglected—a physician's advice should be at once obtained.

XV. In cases of suspected pregnancy, either of mother or nurse, the child should be weaned at once.

XVI. A child ought to be vaccinated after the fifth month, or earlier should small-pox be prevalent.

---

QUACK ADVERTISEMENTS.—The most despicable of all literary frauds and liars are those employés of nostrum mongers who assume the character of physicians and write down the profession for the purpose of writing up quackery. Of course this is done anonymously, for no respectable physician could make such a fool of himself. It is a part of the quack-medicine machinery. The inventor of a preparation to be put on the market as a new and valuable discovery, employs some smart scribbler who is otherwise a miserable hack, to puff it. For this purpose no fiction is too gross and mendacious. Assuming the character of a respectable physician of great experience, the hired penman throws himself soul and body into the service of his master and stops at no falsehood which his skill in that line may suggest. Nor has he any difficulty to get a hearing before the public. He knows that newspapers in general have their price, and that "religious" periodicals are not above the captivating influence of money. We have just observed in a weekly religious journal published in the East, a long article of the kind referred to, in which the writer represents himself as a "reporter" giving the experience of an old and well-known doctor, who was cured of some disease (not of lying) by a certain quack "kidney cure," when his professional brethren had failed to relieve him. Of course the "Doctor" was manufactured in the brain of the reporter, and yet the story will gain credence with a large per cent. of readers, particularly as it appears in a religious paper, and not in the usual form of an advertisement.

There is greatly needed a sense of personal responsibility on the part of journalists in regard to the advertisements which they publish. At present this is entirely wanting. There is no one to stand between the conductors of a journal and the public. Not only are editors and managers irresponsible in this respect, but their direct endorsement of the most palpable falsehoods is often purchasable with money. The agent of the unprincipled nostrum-monger or abortionist goes to the newspaper manager with a proposition substantially as follows: "I want you to publish for me this budget of lies—forged certificates of cure, disguised abortion notices, and so forth. I want it inserted, not as an advertisement, but under another head, so that people will think the editors have written or indorsed it. I want to buy, not only the use of your columns, but, for the time being, that of the names, character and souls of all the editors and others concerned with your paper. What is your charge?" The representative of the press answers: "Our people don't much like to father such a job as that. One of them is a preacher, you know, and they are nearly all strictly religious. We shall have to charge you just double price for putting that stuff in as you want it. Our men can't sell their consciences for nothing." And so the bargain is ended.

We do not pretend to say that this is the literal method of transacting the business, nor that every journalist is guilty of it. But the picture is not much overdrawn, and the practice is injurious and disgraceful and ought to be abandoned.—*Pacific Medical and Surgical Journal.*

---

E. PASTEUR'S EXPERIMENTS ON CHARBON.—M. Pasteur has now an opportunity of practically testing the truth of his theory as to the protection of animals from charbon by their inoculation with the artificially cultivated virus of that disease. On the 5th ultimo, the farm of a veterinary surgeon at Pouilly-le-Fort, and sixty sheep were placed at M. Pasteur's disposal. Ten of these sheep were left untouched, in order that they might later on serve for comparison. Of the remaining fifty, twenty-five were marked with a hole in their ears, and were inoculated, the first time on the 5th of May, and the second on the 17th. On May 31, none of the inoculated sheep had lost fat, or spirits, or appetite. On May 31, the fifty sheep were taken, without distinction, and inoculated with the strongest virus. M. Pasteur predicted that, on the 2d instant, the twenty-five sheep not inoculated would be dead, and that the inoculated animals would show no symptoms of sickness ; and accordingly, on that day, a number of spectators, including the President of the Agricultural Society of Melun, the Prefect of the Department, and the Director of Agricultural Matters at the Ministry of Agriculture and Commerce, assembled to witness the result. The prophecy of M. Pasteur was exactly fulfilled. At two o'clock twenty-three of the sheep which had not been inoculated were dead. At three o'clock the twenty-fourth died, and the twenty-fifth an hour later. The twenty-five inoculated animals were quite sound, and in perfect health. Only one of them was feverish ; and the fever, which was caused by the animal having designedly been inoculated with too strong a dose of the virus, speedily disappeared. The twenty-five carcases have been buried in a fixed spot; and, on the infected grass which will grow over it, experiments are to be made with inoculated and non-inoculated sheep. M. Pasteur's discoveries in this direction are being amply corroborated by English experience, and, if the matter be properly taken up, the agricultural interest will have one less difficulty to contend against, since it ought to be practicable to protect herds against charbon much in the same way that human beings can protect themselves against small-pox by vaccination.

---

INTESTINAL RESECTION.—Professor Czerny communicates (*Berl. Klin. Woch.*, No. 45, 1880), three cases of intestinal resection which he has performed. In two cases, the gangreneous coil of intestine in a strangulated hernia was resected ; and, in the third case, a malignant tumor of the colon was removed. In one of the first two cases, the patient recovered perfectly without fever or reaction of any sort ; in the second, the already pulseless patient died during operation in a fit of fæcal vomiting. The third case was the most remarkable. A female, 47 years old, with a tumor in the abdomen, diagnosed as intestinal cancer, had vomiting and diarrhœa. On operation, there was found a large tumor of the tranverse colon, firmly attached to a coil of the sigmoid flexure. Part of the sigmoid flexure was first resected, the ends being brought together with thirty-three sutures ; then a large piece of the colon was removed, and the ends united by twenty-six sutures. Lastly, a wedge-shaped piece of the meso-colon was removed, also necessitating ligatures. A drainage tube was inserted, and the wound in the abdominal wall closed by means of four deep and eight superficial sutures. The patient recovered, and was living half a year after the operation, although, from the infiltration of the mesenteric glands, a recurrence is inevitable. Dr. Czerny proceeds as follows : The intestine being compressed above and below, the diseased part is removed along with the corresponding wedge of the mesentery. The ends are now disinfected, and a double row of carbolized silk sutures applied. Those in the first row include about a twelfth of an inch of the serous and muscular layers of each end of the intestine, and are about a twelfth of an inch apart. A second row of sutures, about a fifth of an inch apart, are applied over and partly including the first. The intestine is then returned into the abdomen, and antiseptic dressing applied.

---

QUEBRACHO.—Dr. Stewart (*Canada Medical Journal*), says: First introduced by Penzoldt, it has been found to be a decided palliative in many cases of dyspnœa. It is especially valuable in the dyspnœa of emphysema and chronic bronchitis. In dyspnea depending upon valvular insufficiency its value is questionable. Penzoldt has lately experimented with an alkaloid which is obtained from this bark. It is called *aspidospermin*, and occurs in small, white, prismatic crystals. Ten milligrams of a one-per-cent. solution of this alkaloid caused complete motor paralysis in frogs, with marked reduction of both pulse and respiration. Penzoldt administered it to eight patients suffering from dyspnœa due to various causes. In all there was considerable relief; in two this was very marked. According to Penzoldt, it has an undoubted influence over dyspnœa, especially that attending emphysema, but is inferior to the quebracho itself. Dr. Picot, of Carlsruhe, used a tincture of the quebracho bark while doing some mountain climbing, with the result that he could climb with much greater ease and comfort. He has also used it in patients suffering from dyspnœa, and found it act well. In the same number of the *Berl. Klin. Woch.* Berthold recommends it highly. Flint has used it with success also.—*Louisville Medical News.*

---

CONSTIPATION IN INFANTS.—The following are some of the remedies found useful by Dr. D. H. Cullimore (*London Lancet*): 1. A pellet of butter and brown sugar or treacle every morning fasting, or a little raspberry jam. 2. The morning insertion into the rectum of a conical piece of white curd soap about two inches and a half long. It must be first dipped in warm water, held *in situ* for five minutes, and withdrawn. 3. Daily friction over the body, from the right iliac region along the course of the gut, with a little salad oil. In India I have used cocoanut oil advantageously. Cod-liver oil is very useful when its smell is not objected to. *En passant*, I may say that I have at present under my care a girl of fifteen who for a couple of months has suffered from obstinate constipation. She has lately had typhoid. Both mild and strong purgatives were ineffectual, and it has now yielded to cod-liver oil friction. Assiduous friction, without any unguent, is often equally useful. Patience, however, is necessary. A teaspoonful of fluid magnesia in the food is a good plan. Tomato jelly is sometimes used in India with benefit. Whatever plan may be adopted, it is well to supplement it with the internal administration of half a drop of tincture of nux vomica three times a day; a quarter of a drop is sometimes sufficient. Minute doses of sulphur also answer well.—*Louisville Medical News.*

